# On Pepsin, and Its Chemical and Physiological Properties

**Published:** 1857-12

**Authors:** 


*On Pepsin, and its Chemical and Physiological Praperties.* (Bulletin de Thérapeutique, January, 1857.)

In an analysis of a paper read lately by M. Boudault, to the Société de Pharmacie, of Paris, the author, after discussing the general properties of pepsin, proceeds to make the following remarks upon that substance employed as a medicine:

Its administration presents some rather considerable difficulties, in consequence of its liability to alteration when the vessel which contains it has been open. Besides this, its origin, its viscosity, and its disagreeable smell were so many motives for disliking it on the part of the patient. It was necessary, then, to find a method of transforming it without injuring its medical action. It was to be feared, in associating it with an inert substance, that the latter would experience a kind of digestion, or would act upon the pepsin as a ferment. It was necessary, besides, that this substance should be sufficiently hygrometric to absorb the humidity of the pepsin, and not to attract, in addition, the humidity of the air. Sugar was one of the substances with which it appeared most easy to associate pepsin; but at the end of some days the cane-sugar is transformed, under its influence, into glucose, and afterwards into lactic acid, for here the pepsin acts as a true ferment. Starch dried at 100 degrees (Cent.) has given to M. Boudault the most favorable results. Starch, which has the property of not injuring the digestion, forms with pepsin a pulverulent matter, the odor of which is very weak, and the taste partly disguised. This powder is preserved very well in well-stopped bottles, and time does not modify in any way its physiological properties. Under this form, pepsin may be mixed with a number of medicinal substances which do not at all modify its therapeutic action; thus, with hydrochlorate of morphia, to relieve violent pains of the stomach; with strychnine, to stimulate the peristaltic movements of this organ; with nitrate of bismuth, lactate of iron, carbonate of iron, iodide of iron, and other similar preparations. It is very efficacious in dyspepsia, and in all cases of difficult digestion which generally follow the convalescence from serious or chronic diseases; and it has been found a powerful digestive agent in cases of consumption caused by insufficient food. Pepsin is administered in the first spoonful of soup, or even before meals, wrapped up in a wafer; and precaution must be taken not to eat immediately afterwards food which is at a higher temperature than 45 deg., for then the digestive properties of pepsin would be destroyed. It is employed in the acid or in the neutral state. In the acid state it takes the place of the gastric juice, when the latter is not formed in sufficient quantity in certain morbid affections; in the neutral state —that is to say, feebly acidulated—in cases where the stomach contains too great a quantity of acid. It may be shown that chemical or artificial pepsin may very well take the place of the gastric juice, and may be considered among one of our most heroic remedies.—*Ibid.*

